# Beyond single cut-off values in ultrasound: limitations and implications for context-aware radiology reporting

**DOI:** 10.1186/s13244-026-02371-9

**Published:** 2026-08-17

**Authors:** Isabelle Schöpper, Martha Dohna, Paul Martin Bansmann

**Affiliations:** 1 Department of Diagnostic and Interventional Radiology, Hospital Porz am Rhein, Cologne, Germany; 2 Department of Diagnostic and Interventional Radiology, Paediatric Radiology, University Hospital Bonn, Bonn, Germany

**Keywords:** Continuous biomarkers, Cut-off values, Quantitative ultrasound, Radiology reporting, Ultrasound variability

## Abstract

**Abstract:**

Quantitative ultrasound frequently relies on single cut-off values to dichotomize continuous measurements into “normal” and “abnormal.” Although embedded in workflows and guidelines, their biological plausibility and their external validity may be limited in heterogeneous clinical populations and across different technical settings. This review evaluates the conceptual foundations, clinical implications, and relevance of single cut-off values for contemporary radiology reporting. A critical review of studies, consensus statements, and international guidelines published between 1991 and February 2026 was conducted, predominantly via PubMed/MEDLINE, with emphasis on recent and clinically relevant evidence. Research on diagnostic accuracy, population-based studies, and methodological investigations was qualitatively synthesized to assess the biological, technical, and clinical limitations of fixed cut-off values in ultrasound. As this article was not designed as a systematic review, no formal meta-analysis or PRISMA flowchart was included. Most quantitative ultrasound parameters reflect continuous biological processes influenced by patient characteristics, technical factors, and operator variability. Dichotomization may lead to information loss, misclassification, and reduced generalizability, particularly near decision boundaries (“gray zones”). Evidence from vascular ultrasound, elastography, and organ volumetry supports continuous risk gradients rather than discrete biological thresholds. Reliance on single cut-off values in ultrasound largely reflects historical and methodological convention rather than biological discontinuities. Context-aware interpretation strategies, including percentile-based references, multivariable risk models, probabilistic reporting, and longitudinal assessment, may better reflect biological variability and diagnostic uncertainty. Thresholds may therefore evolve from rigid decision boundaries toward context-sensitive reference points within modern reporting frameworks, thereby helping radiologists communicate diagnostic uncertainty more transparently.

**Critical relevance:**

This review critically assesses fixed ultrasound cut-offs and supports context-aware reporting to reduce misclassification near decision boundaries and improve individualized, clinically meaningful radiology interpretation.

**Key Points:**

- Single cut-off values remain widely used in quantitative ultrasound, despite uncertainty about their biological validity, diagnostic reliability, and generalizability in clinical practice.
- Fixed ultrasound thresholds remain clinically useful but may lead to misclassification when biological variability, technical factors, and clinical context are not accounted for.

**Graphical Abstract:**

**Figure:**
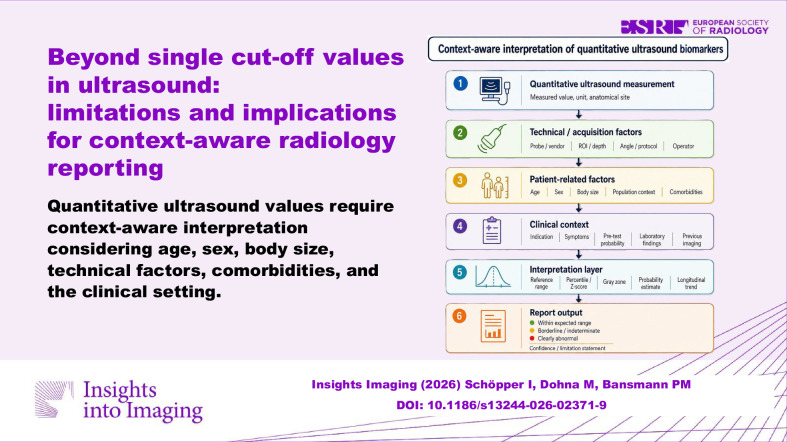

## Introduction

Ultrasound (US) is among the most widely used imaging modalities worldwide, valued for accessibility, real-time capability, and broad clinical applicability. Advances in transducer technology, signal processing, contrast enhancement, elastography, and data analytics have increasingly enabled the development of quantitative imaging biomarkers. Although standardization and AI-assisted tools may improve measurement reproducibility [1], interpretation of many ultrasound-derived parameters still relies on single cut-off values that dichotomize continuous measurements into “normal” and “abnormal”.

Reference intervals are often defined as the central 95% of a healthy reference population (2.5th–97.5th percentiles). Thus, approximately 5% of healthy individuals fall outside this interval without necessarily having disease [2]. Fixed thresholds are perceived as objective and easy to communicate, particularly in screening, structured workflows, and guideline pathways.

However, this simplicity conceals conceptual and practical limitations. Many reference values were established decades ago in cohorts that differ from contemporary populations in terms of higher age, higher rates of overweight and obesity, demographic shifts, sex distribution and body composition [3, 4]. As a consequence, older thresholds may inflate “pathological” findings that partially reflect population shifts. At the same time, normalization of widespread conditions such as obesity must not obscure their pathophysiological relevance and cardiovascular implications [5].

Beyond demographic shifts, inter-individual biological variability and measurement-related factors challenge the use of rigid cut-offs. Many biomarkers are context-sensitive and influenced by age, sex, body size, hemodynamic state, comorbidities, and technical settings [6, 7]. In increasingly diverse populations, thresholds derived from relatively homogeneous reference cohorts may be less generalizable across clinical settings, given differences in anthropometry, risk profiles, and disease prevalence [8–12].

Contemporary methodological literature supports interpreting continuous biomarkers as continuous or model-based predictors rather than as dichotomous variables. Dichotomization of continuous biomarkers has been criticized for information loss, reduced statistical efficiency, and unstable decision boundaries [13–16]. Ultrasound occupies a critical position in this debate, given its inherent variability [17, 18] but also its unique strengths in dynamic and repeatable assessment [19, 20].

This narrative review examines the origins, methodological foundations, and clinical consequences of single cut-off values in quantitative ultrasound. It further proposes a framework for context-aware interpretation that integrates biological variability, technical uncertainty, and clinical context. This approach aims to support radiologists in moving beyond rigid dichotomous classification toward more clinically meaningful, probability-informed reporting.

## Review approach and literature selection

This critical review aimed to analyze methodological, biological, technical, and clinical limitations associated with single cut-off values in quantitative ultrasound. Publications from 1991 through February 2026 were considered, with emphasis on recent and clinically relevant evidence. A structured PubMed/MEDLINE search was performed using combinations of the terms quantitative ultrasound, cut-off/threshold, dichotomization, reference values, diagnostic accuracy, risk prediction, continuous biomarkers, ultrasound variability, interoperability, and selected applications (e.g., elastography, vascular ultrasound, intima-media thickness). The review focused primarily on adult ultrasound applications. Pediatric or adolescent reference studies were considered only when they illustrated broader methodological issues related to reference values and threshold-based interpretation.

Publications were selected based on their relevance to the conceptual, methodological, or clinical implications of fixed cut-off values.

Approximately 200 publications were screened. Included publication types comprised original observational and diagnostic studies, reviews and meta-analyses, methodological/statistical papers, consensus statements, guidelines and reporting/scientific statements. Case reports and individual randomized interventional trials were not included as primary source articles. Reference lists of relevant articles were additionally screened to identify further sources.

Studies and documents were prioritized if they fulfilled at least one of the following criteria: (1) they addressed methodological limitations of dichotomizing continuous biomarkers; (2) they provided empirical evidence for variability, overlap, or uncertainty of quantitative ultrasound measurements; (3) they represented consensus statements or guidelines relevant to clinical ultrasound reporting; (4) they illustrated clinically relevant examples in which fixed thresholds are widely used; or (5) they discussed probabilistic, percentile-based, multivariable, or longitudinal interpretation strategies.

The selected clinical examples, including carotid intima-media thickness (CIMT), liver elastography, organ volumetry, carotid velocity criteria, renal resistive index (RI), common bile duct (CBD) diameter, and abdominal aortic diameter, were chosen because they represent common ultrasound applications in which quantitative thresholds are frequently used in clinical practice and where the balance between pragmatic utility and biological or technical limitations can be critically examined.

English-language publications were primarily considered. No formal meta-analysis or structured risk-of-bias assessment was performed, consistent with the critical review design. As this article was not designed as a systematic review, no PRISMA flowchart was included. As a narrative critical review, study selection was not exhaustive, and selection bias cannot be excluded. The last search was conducted on February 15, 2026.

## Why single cut-off values emerged in ultrasound diagnostics

Single cut-off values became popular because they were practical, facilitated reporting, and supported decision-making in settings where early quantitative ultrasound measurements were variable and operator-dependent. Standardization of protocols and images enabled broader comparability. Binary classification (“normal vs abnormal”) fits well into clinical pathways and structured reporting [21, 22].

This approach was reinforced by conventional diagnostic accuracy methods, which define thresholds to optimize sensitivity and specificity. However, prediction modeling shows that dichotomizing continuous variables reduces information, may impair calibration, and can yield unstable decision limits when populations, spectra, or prevalence differ [23, 24]. Many thresholds were established under controlled conditions with selected cohorts, often predominantly male [25–28], limiting transferability to real-world practice with heterogeneous equipment and diverse patients, an issue acknowledged in World Federation for Ultrasound in Medicine and Biology (WFUMB) and Society of Radiologists in Ultrasound (SRU) documents [21, 29].

Thresholds remain useful as pragmatic reference points for communication and rule-in/rule-out decisions. Their limitation lies in use as deterministic boundaries without accounting for uncertainty, variability, and clinical context. Decision-analytic frameworks emphasize that clinical usefulness depends on pre-test probability, patient context, and downstream consequences, not solely on sensitivity/specificity [30, 31].

## Biological continuity versus dichotomous thresholds

Most ultrasound-derived parameters represent continuous biological processes rather than discrete disease states. Organ size, stiffness, and flow parameters vary physiologically with age, sex, body composition, and hemodynamic conditions, challenging universal cut-offs [32, 33].

In vascular ultrasound, carotid intima-media thickness (IMT) illustrates this: meta-analyses show continuous associations with cardiovascular disease outcomes, without a clear biological threshold. Therefore, IMT may be better incorporated into multivariable cardiovascular risk estimation (for cardiovascular disease) rather than used as a standalone dichotomous criterion [34, 35].

Liver stiffness is another example. Although cut-offs for fibrosis staging are frequently used, values overlap between histologic stages, and confounders such as inflammation, cholestasis, or congestion can increase stiffness independent of fibrosis progression [21, 36]. Guideline-based interpretation, therefore, often relies on ranges rather than a single universal threshold. For example, transient elastography values below 8–10 kPa may help rule out compensated advanced chronic liver disease, whereas values above 12–15 kPa may support rule-in, with intermediate values requiring further assessment [33]. These limitations support the concept of “gray zones,” where uncertainty peaks near decision boundaries. Consensus statements recommend caution around thresholds and emphasize quality criteria and clinical context [36, 37]. Accordingly, liver stiffness values close to commonly used thresholds should be reported with explicit uncertainty rather than being translated into deterministic fibrosis stages.

## Technical variability, measurement uncertainty, and clinical context

Quantitative ultrasound measurements are sensitive to probe characteristics, depth, ROI placement, patient positioning, breathing phase, and post-processing, generating clinically relevant variability [22, 32]. In elastography, inter-system and inter-vendor variability are widely recognized, and values may not be interchangeable across platforms. Therefore, thresholds may not transfer reliably [21, 36].

Operator dependence further amplifies dispersion. Interobserver variability has been reported for hepatic steatosis and thyroid nodule measurements [38, 39]. Patient factors also matter: organ sizes vary with sex, age, and body size, and male-derived thresholds may misclassify women [25]. For example, spleen and liver sizes are larger in men [40], spleen size increases, and kidney length decreases with age [41]. Additionally, body habitus affects comparability [42, 43]. Misclassification is most likely near cut-offs, where small differences can flip categories and trigger downstream testing [36, 37].

Measurement uncertainty is inherent to ultrasound, and small variations near thresholds may change categorization. Finally, diagnostic implications depend on indication, disease prevalence, and pre-test probability [30, 31]. Longitudinal assessment underscores context dependence. For chronic liver disease, guidelines recommend combined strategies and clinical context, and repeated measurements can be more informative than single-time-point classification [33, 36].

## Clinical examples where rigid thresholds are limited

### Carotid intima-media thickness (CIMT)

Meta-analyses by Den Ruijter and Willeit support continuous associations with cardiovascular risk rather than a distinct “abnormality threshold”. In the USE-IMT (“Usefulness of Carotid Intima-media Thickness”) individual participant data meta-analysis, each 0.1 mm higher common CIMT was associated with increased cardiovascular risk (e.g., 10-year risk of first-time myocardial infarction or stroke), but the addition of CIMT to the Framingham Risk Score only marginally improved discrimination and reclassification. This supports CIMT as a continuous risk marker rather than a binary abnormality threshold [34, 35]. In addition, Randrianarisoa et al demonstrated that traditional thresholds (e.g., 1 mm) may not reflect contemporary distributions. Values vary by age and sex [44].

### Organ volumes (e.g., thyroid, ovary)

Physiological variability yields broad normal ranges that overlap with early pathology, increasing the risk of overdiagnosis if isolated cut-offs are applied. For ovarian volume, age-adjusted reference curves are recommended [45]. For thyroid volume, fixed limits (e.g., 18 mL women, 25 mL men) irrespective of age may be overly coarse [46] and could misclassify subgroups. Percentile-based references accounting for population and iodine status may be more appropriate [47]. Recent work suggests additional refinement may be needed [48].

### Vascular ultrasound velocity thresholds (carotid stenosis)

Consensus criteria frequently use peak systolic velocity (PSV) cut-offs [49]. However, PSV is sensitive to protocol and equipment (e.g., angle correction, sampling location, Doppler settings), limiting its transferability. Interobserver variability in PSV determination has been reported even among experienced examiners [50]. These characteristics make rigid thresholds vulnerable near category boundaries.

## Where cut-offs remain clinically useful

Despite limitations, several ultrasound parameters have robust normal ranges that remain clinically useful when interpreted in context. This review does not argue for abandoning thresholds in quantitative ultrasound. Rather, single cut-off values should be understood as pragmatic reference points that support standardized communication and clinical decision-making, including rule-in/rule-out assessment, screening, surveillance, and triage, particularly when well established and interpreted alongside clinical context. These cut-offs often function best as decision support, not as absolute truth. Several examples illustrate this balanced role of cut-offs:

### Renal resistive index (RI)

The RI increases with age from 0.58 in young adults to 0.70–0.72 in elderly individuals [51, 52]. While RI may serve as an early marker of renal disease in younger patients, specificity decreases in older individuals due to vascular aging and stiffness [53]. Side-to-side comparison supports detection of unilateral pathology [52]. Thus, RI remains clinically useful, but its interpretation should take into account age, vascular status, laterality, and the clinical indication.

### Common bile duct (CBD) diameter

CBD diameter thresholds ( ≤ 6 mm; ≤ 10 mm after cholecystectomy) remain commonly used [54–57]. Their practical value lies in providing a simple reference for deciding whether further clinical or laboratory correlation is needed. Although diameter can increase with age, average values often remain near these ranges [58]. Therefore, mild threshold exceedance should not automatically be interpreted as obstruction, but should be assessed in relation to symptoms, liver enzymes, cholecystectomy status, and prior imaging when available.

### Abdominal aortic diameter

A diameter < 30 mm is generally considered normal and defines abdominal aortic aneurysm [59]. This is an example of a threshold with clear practical value in screening and surveillance because it is simple, widely accepted, and clinically actionable. However, women have smaller aortic diameters than men [60, 61]. Uniform thresholds are therefore pragmatic but may not fully capture sex-specific risk. Accordingly, even established thresholds may require contextual interpretation when patient characteristics influence baseline anatomy or risk.

Taken together, these examples show that the clinical value of cut-offs depends on their intended use. Thresholds are most helpful when they provide a common reporting language, support established diagnostic or surveillance pathways or indicate the need for further clinical correlation. Their limitations arise when they are interpreted as absolute biological boundaries rather than standardized reference points that require contextual interpretation.

## Toward context-aware ultrasound reporting

This framework emphasizes that quantitative ultrasound measurements cannot be fully interpreted without contextual integration, including patient characteristics (age, sex, body size), technical/operator factors, and clinical history (comorbidities, interventions). Examples include transient organ enlargement after acute infection or compensatory hypertrophy after unilateral nephrectomy, which may affect measurements and lead to misclassification if not considered. Moving beyond single cut-offs does not mean abandoning quantitative interpretation, but using measured values as context-sensitive reference points.

### Percentile-based reference values and *Z*-scores

**Fig. 1:**
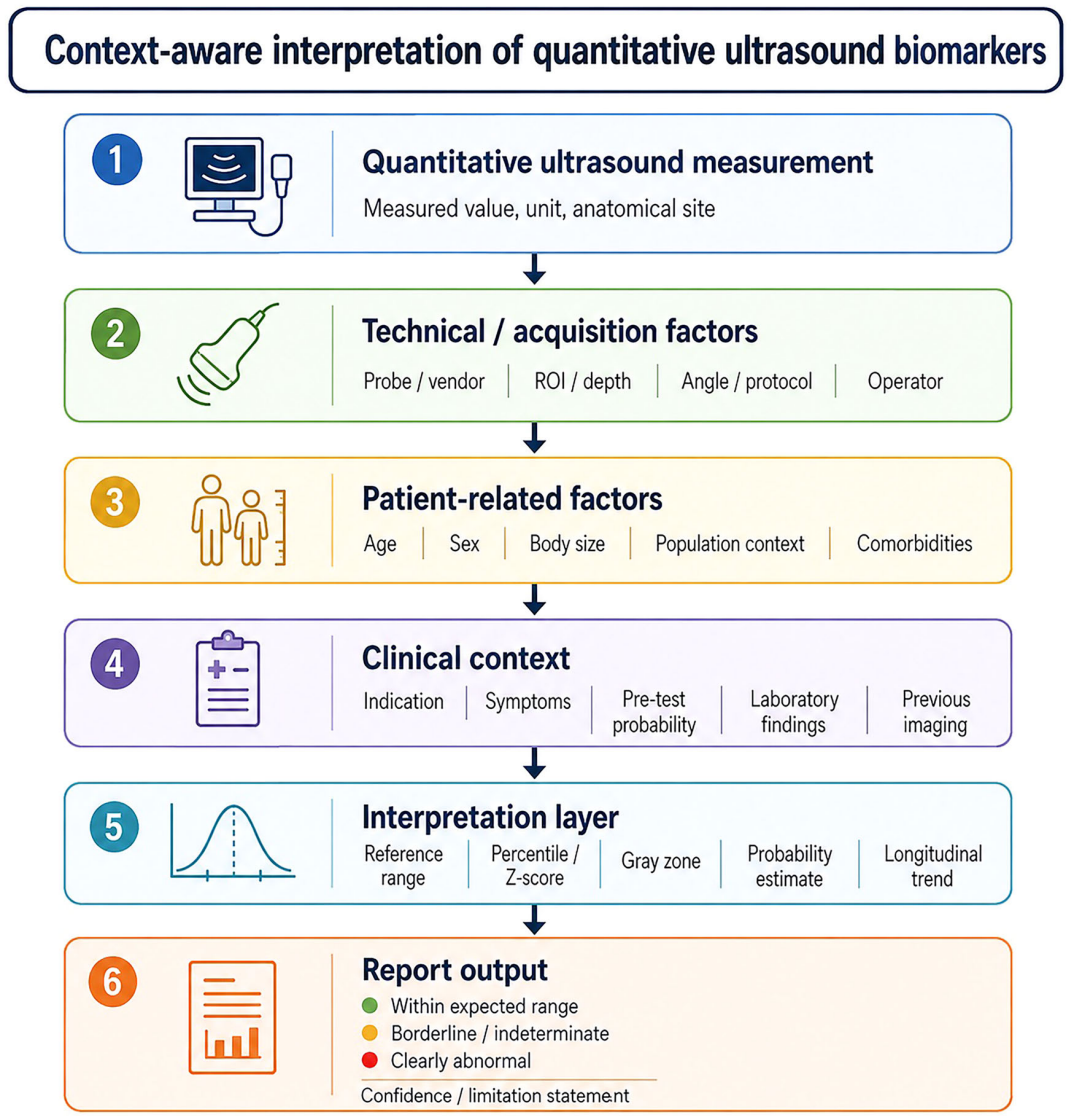
Stepwise framework for integrating technical, patient-related, and clinical context into quantitative ultrasound reporting

These contextualize measurements relative to age-, sex-, and body size-adjusted distributions, reducing biologically implausible universal thresholds [32, 33]. Kidney length illustrates this: a 9.5 cm kidney may be normal in a small elderly woman but unusually small in a tall young man. Renal dimensions correlate with habitus and sex [62]. Universal thresholds (e.g., “< 10 cm abnormal”) therefore risk misclassification. *Z*-scores quantify deviation from the reference mean [63] and may improve comparability [13, 64], particularly when anatomical variants (e.g., solitary kidney) are present (Fig. 1).

### Probabilistic reporting and gray zones

Probabilistic reporting aligns ultrasound communication with evidence-based medicine by expressing likelihood/confidence rather than deterministic labels. Decision curve analysis can evaluate whether probabilistic or model-based strategies improve clinical net benefit across relevant threshold probabilities [30, 31].

**Fig. 2:**
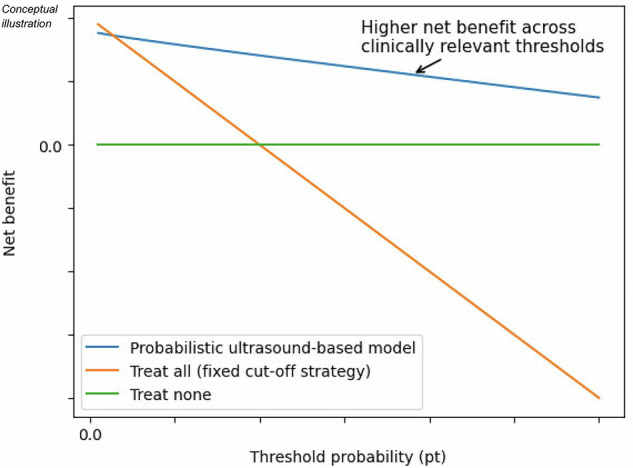
Conceptual illustration of decision-curve principles applied to quantitative ultrasound reporting. The curves are illustrative only and not based on original patient-level data or a formal decision-curve analysis. The figure is intended to demonstrate how probabilistic interpretation could be evaluated against binary threshold-based strategies in future studies

A practical example is carotid duplex ultrasound: PSV cut-offs are used to categorize stenosis severity, yet values near thresholds are sensitive to variability and clinical context. Likelihood-based reporting (e.g., low/intermediate/high probability of ≥ 50% or ≥ 70% stenosis) can better reflect uncertainty. Decision curve analysis can test whether such strategies reduce missed clinically relevant stenoses and unnecessary downstream imaging compared with a single PSV cut-off [65]. Figure 2 provides a conceptual illustration, not an original decision-curve analysis.

In decision curve analysis, a probabilistic strategy can be compared with “treat none” and “treat all.” If the model yields higher net benefit across clinically relevant threshold probabilities, it supports risk-based reporting rather than rigid cut-offs.

### Longitudinal assessment

Trend-focused interpretation reduces the impact of single-measurement variability and is particularly relevant for chronic disease monitoring [33]. In practice, documenting change over time can be more actionable than labeling one value as “abnormal” based on a fixed boundary.

## Practical reporting examples for routine use

A key reason cut-offs persist is their operational simplicity. Rather than removing thresholds, the practical goal is to use them differently: as context-sensitive reference points and prompts for integration.

1. **Report uncertainty explicitly near boundaries (“gray zones”)**. Instead of a deterministic “normal/abnormal” label, use tiered language:

- “within expected range”
- “borderline/indeterminate range”
- “clearly elevated/decreased”

Add a short context statement when appropriate:

- “interpretation limited by suboptimal acoustic window/motion/depth”
- “value may be affected by inflammation/congestion/hemodynamic” (e.g., elastography) [21, 36, 37].

2) **Prefer contextual reference frameworks when available.** For measurements strongly dependent on age/sex/body size (organ size/volume), consider percentile/Z-score framing where feasible [13, 62–64].
3) **Integrate pre-test probability and indication**. Thresholds have a different meaning in screening vs symptomatic patients. Interpretation should reflect indication and prevalence [30, 31].
4) **Use trends for monitoring whenever possible**. For chronic disease, repeated measurements under comparable conditions can be more informative than classifying single values [33].
5) **Example wording:** Examples of how conventional threshold-based statements can be reframed into context-aware reporting language are provided in Table 1. Importantly, definitive reporting remains appropriate for clear findings. Context-aware wording is particularly useful for borderline measurements, technical limitations, or relevant biological and clinical modifiers (Table 1).

**Table 1:** Examples of conventional threshold-based and context-aware reporting in quantitative ultrasound

Clinical examination	Threshold-based wording	Context-aware wording
Carotid stenosis	“PSV indicates ≥ 50% stenosis.”	“PSV suggests an intermediate probability of ≥ 50% carotid stenosis. Interpretation assumes a standard duplex protocol and should consider possible technical measurement bias. Clinical relevance depends on symptoms, vascular risk factors, and prior imaging” [49, 50].
Kidney length	“Kidney length < 9 cm, abnormal.”	“Kidney length (8.5 cm) is below the commonly used reference values. Interpretation should consider age, sex, body size, laterality, and previous measurements as well as measurement uncertainty. In a 20-year-old woman with a BMI of 19.0, this may be within an expected range.”
Common bile duct diameter	“CBD diameter is abnormal.”	“CBD diameter of 7 mm is mildly enlarged relative to standard thresholds. Interpretation should also consider age, symptoms, liver enzymes, and interval change.” **or** “CBD is dilated, measuring 11 mm. In the post-cholecystectomy setting not necessarily pathological. Clinical correlation is recommended.”
Abdominal aortic diameter	“Aortic aneurysm with a diameter > 30 mm.”	“Aortic diameter of 31 mm is mildly above the conventional aneurysm threshold. Interpretation should consider sex, body size, risk factors, and the indication for screening or follow-up. In this male patient with a tortuous aortic course, the finding may be of limited pathological significance.”
Elastography	“Liver stiffness above the threshold, elevated.”	“Liver stiffness is elevated. Given overlap across fibrosis stages and potential confounders, it should be interpreted in the clinical context” [33, 36, 37].

## Role and limitations of artificial intelligence

Artificial intelligence (AI) in quantitative ultrasound has two main roles. First, it can automate measurements by identifying landmarks, segmenting organs, and extracting standardized values (e.g., organ or lesion volumetry), which may reduce operator variability. AI may also support more standardized assessment of image-derived features, such as liver echogenicity, for which prior work has noted limited correlation between steatosis and visually perceived brightness due to subjectivity [66–68].

Second, AI can support risk prediction by combining ultrasound features with clinical variables to estimate individualized probabilities instead of applying a single cut-off [69]. However, such models require external validation, calibration assessment, and transparent reporting before routine clinical use [70], consistent with expectations in other modalities [71].

A key challenge is dataset shift and vendor dependency: differences in hardware, presets, and acquisition protocols can reduce generalizability outside the development environment [72]. Reporting frameworks such as transparent reporting of a multivariable prediction model for individual prognosis or diagnosis using artificial intelligence (TRIPOD-AI) and risk-of-bias tools, including the prediction model risk of bias assessment tool for artificial intelligence (PROBAST-AI), therefore emphasize applicability, external validation, and uncertainty communication [73]. Importantly, AI-based contextualization should not be interpreted as eliminating uncertainty. Rather, uncertainty may shift from manual measurement variability to model calibration, external validity, dataset shift, and workflow integration. Prospective multicenter validation across vendors, acquisition protocols, and patient populations is therefore essential before AI-supported probabilistic reporting can be implemented in routine ultrasound.

## Strengths and limitations

This narrative review has several strengths. It integrates methodological, biological, and clinical perspectives on cut-off values in quantitative ultrasound and links these concepts to contemporary radiology reporting frameworks. By combining evidence from diagnostic accuracy research, guideline documents, and methodological work on probabilistic reporting and AI-assisted interpretation, this review provides a conceptual framework that may support more context-aware and clinically meaningful ultrasound reporting.

Several limitations should also be acknowledged. It provides a concept-driven synthesis rather than a systematic screening and appraisal process. No meta-analysis or structured risk-of-bias assessment was performed. Because this was a critical review, study selection was not exhaustive and may be affected by selection bias, as examples were chosen for conceptual relevance and clinical illustrative value. The aim was not to provide pooled estimates of diagnostic accuracy, but to synthesize representative methodological and clinical evidence relevant to context-aware ultrasound reporting. The focus is on adult ultrasound and selected domains with established cut-offs. Pediatric, obstetric, and highly specialized settings may involve different trade-offs. International guidelines and consensus documents are referenced, but local implementation varies across institutions, vendors, and healthcare systems. Many classic reference values were derived in homogeneous cohorts without systematic ethnic stratification. For example, normative renal dimensions were derived from adult volunteers in a Danish cohort under controlled conditions [62]. Later studies show that organ dimensions vary with anthropometric and population characteristics [47], supporting the need for validation or recalibration in contemporary, diverse populations.

AI-supported approaches are discussed as emerging tools. While promising, prospective validation, standardization, and workflow integration remain prerequisites for clinical routine [70, 73].

## Outlook

Future developments may accelerate a shift toward individualized, risk-based interpretation. AI can automate measurements and help integrate patient and device factors into adaptive reference models. Structured reporting may therefore incorporate risk categories and certainty levels rather than binary labels [74, 75], operationalized through tiered likelihood language, brief context statements, and trend-based interpretation [30, 31, 76].

Table 2 summarizes strategies that extend beyond a single cut-off value. Binary thresholds remain practical for some rule-in/rule-out decisions but are prone to misclassification near boundaries. Percentile/Z-score approaches adjust for age, sex, and body size. Multivariable models integrate imaging and clinical data to generate individualized risk estimates. Probabilistic tiers communicate uncertainty in borderline ranges. Longitudinal trend analysis emphasizes change over time. Hybrid strategies combine thresholds with contextual and temporal information and may be most practical in routine ultrasound practice.

**Table 2:** Interpretation strategies beyond single cut-offs in quantitative ultrasound

Strategy	Key idea	Main advantage	Main limitation	Typical clinical application
Binary threshold	Single numerical cut-off as a reference point for standardized decision-making	Simple and standardized	Misclassification risk near cut-off	Screening, triage, guideline pathways, rule-in/rule-out decisions
Percentiles/*Z*-scores	Adjustment for age, sex, and body size	Reflects biological variability	Requires reference datasets	Organ volumetry, pediatrics
Multivariable model	Integration of imaging and clinical data	Personalized probability	Needs validation and integration	Risk stratification
Probabilistic classification	Categorization into likelihood ranges	Communicates uncertainty	Requires standardized reporting language	Borderline values
Longitudinal trend	Assessment of change over time	Reduces the effect of variability	Requires repeated measurements	Disease monitoring, follow-up
Hybrid strategy	A combination of threshold, context, and trend	Most robust interpretation	More complex implementation	Routine clinical practice

These strategies can be applied across various ultrasound applications, including vascular ultrasound, elastography, and organ volumetry

## Conclusion

This review does not advocate abandoning cut-off values in ultrasound. Rather, it argues that thresholds should be interpreted as pragmatic, context-sensitive reference points rather than absolute biological boundaries. Reliance on single cut-off values in ultrasound largely reflects historical and methodological convention rather than biological discontinuities. Many ultrasound-derived parameters are continuous, context-dependent biomarkers whose meaning cannot be fully captured by rigid dichotomization, particularly near decision boundaries where measurement uncertainty and variability are greatest. Technical and operator-related variability further limits universal transferability across vendors and clinical settings. Evidence from diagnostic accuracy research, guideline documents, and methodological work on risk prediction supports more nuanced interpretative frameworks. Approaches such as percentile-based references, multivariable risk models, probabilistic reporting, and longitudinal assessment preserve quantitative information while better reflecting uncertainty and interindividual variability. Accordingly, quantitative ultrasound findings should be interpreted as context-dependent measurements rather than absolute diagnostic boundaries. Despite ongoing standardization and AI-assisted optimization, individualized clinical judgment remains essential to integrate imaging findings into meaningful patient care. This shift toward context-aware interpretation may also support the integration of quantitative ultrasound biomarkers into modern radiology reporting and precision imaging frameworks.

## Data Availability

No new datasets were generated or analyzed during the preparation of this review. All data supporting the conclusions of this article are available in the cited literature.

## Abbreviations

AI: Artificial intelligence
CBD: Common bile duct
CIMT: Carotid intima-media thickness
ESR: European Society of Radiology
IMT: Intima-media thickness
PROBAST-AI: Prediction model risk of bias assessment tool for artificial intelligence
PSV: Peak systolic velocity
RI: Resistive index
SRU: Society of Radiologists in Ultrasound
TRIPOD: Transparent reporting of a multivariable prediction model for individual prognosis or diagnosis
TRIPOD-AI: Transparent reporting of a multivariable prediction model for individual prognosis or diagnosis using artificial intelligence
US: Ultrasound
USE-IMT: Usefulness of carotid intima-media thickness
WFUMB: World Federation for Ultrasound in Medicine and Biology
